# Associations of Multimorbidity With Stroke Severity, Subtype, Premorbid Disability, and Early Mortality: Oxford Vascular Study

**DOI:** 10.1212/WNL.0000000000207479

**Published:** 2023-08-08

**Authors:** Matthew B. Downer, Linxin Li, Samantha Carter, Sally Beebe, Peter M. Rothwell

**Affiliations:** From the Wolfson Centre for the Prevention of Stroke and Dementia, Nuffield Department of Clinical Neurosciences, Wolfson Building–John Radcliffe Hospital, University of Oxford, United Kingdom.

## Abstract

**Background and Objectives:**

Patients with multimorbidity are underrepresented in clinical trials. Inclusion in stroke trials is often limited by exclusion based on premorbid disability, concerns about worse poststroke outcomes in acute treatment trials, and a possibly increased proportion of hemorrhagic vs ischemic stroke in prevention trials. Multimorbidity is associated with an increased mortality after stroke, but it is unclear whether this is driven by an increased stroke severity or is confounded by particular stroke subtypes or premorbid disability. We aimed to determine the independent association of multimorbidity with stroke severity taking account of these main potential confounders.

**Methods:**

In a population-based incidence study (Oxford Vascular Study; 2002–2017), prestroke multimorbidity (Charlson Comorbidity Index [CCI]; unweighted/weighted) in all first-in-study strokes was related to postacute severity (≈24 hours; NIH Stroke Scale [NIHSS]), stroke subtype (hemorrhagic vs ischemic; Trial of Org 10172 in Acute Stroke Treatment [TOAST]), and premorbid disability (modified Rankin scale [mRS] score ≥2) using age-adjusted/sex-adjusted logistic and linear regression models and to 90-day mortality using Cox proportional hazard models.

**Results:**

Among 2,492 patients (mean/SD age = 74.5/13.9 years; 1,216/48.8% male; 2,160/86.7% ischemic strokes; mean/SD NIHSS = 5.7/7.1), 1,402 (56.2%) had at least 1 CCI comorbidity, and 700 (28.1%) had multimorbidity. Although multimorbidity was strongly related to premorbid mRS ≥2 (adjusted odds ratio [aOR] per CCI comorbidity 1.42, 1.31–1.54, *p* < 0.001), and comorbidity burden was crudely associated with an increased severity of ischemic stroke (OR per comorbidity 1.12, 1.01–1.23 for NIHSS 5–9, *p* = 0.027; 1.15, 1.06–1.26 for NIHSS ≥10; *p* = 0.001), no association with severity remained after stratification by TOAST subtype (aOR 1.02, 0.90–1.14, *p* = 0.78 for NIHSS 5–9 vs 0–4; 0.99, 0.91–1.07, *p* = 0.75 for NIHSS ≥10 vs 0–4), or within any individual subtype. The proportion of intracerebral hemorrhage vs ischemic stroke was lower in patients with multimorbidity (aOR per comorbidity 0.80, 0.70–0.92, *p* < 0.001), and multimorbidity was only weakly associated with 90-day mortality after adjustment for age, sex, severity, and premorbid disability (adjusted hazard ratio per comorbidity 1.09, 1.04–1.14, *p* < 0.001). Results were unchanged using the weighted CCI.

**Discussion:**

Multimorbidity is common in patients with stroke and is strongly related to premorbid disability but is not independently associated with an increased ischemic stroke severity. Greater inclusion of patients with multimorbidity is unlikely therefore to undermine the effectiveness of interventions in clinical trials but would increase external validity.

## Introduction

Multimorbidity (often referred to as “multiple long-term conditions”) is common in older individuals,^1^ particularly in patients with stroke,^2^ and prevalence is predicted to rise further with continued population aging.^1,3^ Yet patients with multimorbidity are generally underrepresented in randomized trials,^4^ which may limit the generalizability of results,^5^ and thereby exacerbate the existing tendency to undertreat in routine practice. Exclusion of patients with multimorbidity from acute stroke trials might be driven indirectly by increased premorbid disability, with a cutoff premorbid modified Rankin scale (mRS) score ≥2 excluding over a third of stroke patients older than 65 years and nearly two-thirds older than 85 years^6^ but is also related to concerns about greater severity of stroke and increased early mortality in patients with multimorbidity. Such patients also tend to be excluded from trials of preventive treatments, such as antithrombotic treatment in secondary prevention of stroke,^7^ partly due to a concern about an increased proportion of hemorrhagic vs ischemic recurrent strokes.

It is plausible that there may indeed be adverse impacts of multimorbidity on the severity of stroke, either through effects on pathophysiology and progression of cerebral ischemia and/or on the effectiveness of treatments. Multimorbidity is consistently reported to be associated with an increased mortality after stroke,^8-11^ but it is uncertain whether this association is driven by an increased severity of stroke or is confounded by associations with particular stroke subtypes or with premorbid disability. Previous studies have largely focused on the impact of multimorbidity on overall all-cause mortality in large administrative or hospital datasets,^8-11^ but none have adjusted for premorbid disability or stratified by etiologic subtype. A better understanding of the association of multimorbidity with stroke severity, premorbid disability, and etiologic subtype is therefore required to understand apparent associations with poststroke mortality. We aimed to determine these associations in a population-based stroke incidence study (the Oxford Vascular Study [OXVASC]).

## Methods

The OXVASC is a population-based study of all acute vascular events in a defined population of 94,973 persons registered with approximately 100 primary care physicians working in 9 general practices Oxfordshire, United Kingdom.^12-14^ In the United Kingdom, almost all of the population is registered with a general practitioner (GP), who holds a lifelong record of all medical consultations, investigations, and diagnoses made in either primary or secondary care. Further details on the underlying population have been previously published.^12-14^ The OXVASC has been approved by the local health research ethics committee (OREC A: 05/Q1604/70).

The present sample included all first-in-study strokes that were ascertained from April 1, 2002, to March 31, 2017. Case ascertainment was based on multiple sources, including a daily rapid access transient ischemic attack (TIA)/stroke clinic for patients with suspected TIA or stroke, daily searches of hospital admissions to relevant wards, emergency department attendances, and Bereavement Office records; and monthly searches of death certificates, coroner reports, GP diagnostic codes, and brain/vascular imaging referrals. Further details on the ascertainment methodology used in the OXVASC are reported elsewhere.^12-14^

Patients were seen by study physicians as soon as possible after an index event. At initial assessment, data were collected on clinical and demographic variables, including premorbid disability (mRS),^15^ and prior comorbidities and cross-checked by a review of GP records. All patients also had a detailed clinical examination including an assessment of their postacute phase stroke severity (NIH Stroke Scale [NIHSS]),^16^ usually approximately 24 hours after onset. Data were also collected on brain and vascular imaging, 12-lead electrocardiograph, blood tests, echocardiography, and 5-day ECG monitoring.

If a patient died before a study assessment, information was obtained from records and eye witness accounts if possible. All cases were reviewed by the study's senior neurologist (P.M.R.), and stroke was defined using the World Health Organization criteria^17^ and ischemic stroke subtype classified with the Trial of Org 10172 in Acute Stroke Treatment (TOAST) system.^18^

### Statistical Analyses

Patients with incomplete data (usually <2%) were excluded from analyses and no data were imputed. Continuous data were reported as either mean (SD) or median (interquartile range), as appropriate. Categorical data were presented as counts (percentages). Differences between multimorbidity groups were explored using 1-way analysis of variance, χ^2^, or Kruskal-Wallis test, as appropriate.

Prestroke multimorbidity was quantified using the both the weighted and unweighted versions of the Charlson Comorbidity Index (CCI).^19^ For unweighted analyses, patients were classified as living with no comorbidity (CCI = 0), 1 comorbidity (CCI = 1), or multimorbidity (CCI ≥2) and compared across groups, but regression analyses were also conducted across the full CCI score. Weighted analyses used CCI weights previously validated for stroke outcome studies (eTable 1, links.lww.com/WNL/C911).^10^

Associations between multimorbidity (using unweighted CCI) and stroke severity were analyzed using crude and adjusted logistic regression models for NIHSS 5–9 and ≥10 vs 0–4. Because the longer distribution of the weighted CCI allowed for linear regression analysis, a further set of analyses was also performed using linear regression with the weighted CCI and full ordinal NIHSS score. Analyses were performed including all patients and stratified by TOAST subtype, with subtype-specific associations then pooled by fixed-effects meta-analysis. To explore possible associations between prestroke multimorbidity and subtype, crude and adjusted (age/sex) logistic regression models were used, with cryptogenic stroke used as the reference group for TOAST subtypes. Additional analyses were also performed with stratification by age (younger than 75 years/75 years or older) and sex.

Associations between multimorbidity and premorbid disability were analyzed using crude and adjusted (age/sex) logistic regression models for mRS ≥2 vs 0–1. Associations between multimorbidity and all-cause mortality within 90 days were assessed with Cox proportional hazard models (unadjusted, age/sex adjusted, additionally adjusted for NIHSS and/or mRS, and stratified by TOAST subtype).

We also performed 3 sensitivity analyses. First, a small number of patients had a stroke death in the community or died very shortly after arrival at hospital and therefore did not have NIHSS assessed. To avoid potential bias due to exclusion of such cases, we did a set of analyses examining the within-subtype associations between multimorbidity and severity in which these cases were classified as NIHSS ≥10 and allocated to either the intracerebral hemorrhage category or the TOAST Unknown ischemic stroke subtype. Second, in an attempt to minimize potential bias relating to delayed presentation or initial clinical assessment in patients with multimorbidity, we ran analyses excluding patients who received thrombolysis or thrombectomy. Third, to limit possible bias due to patient or physician decisions to limit investigation/treatment on the association of multimorbidity with 90-day mortality, we repeated analyses after excluding patients with metastatic cancer, hematologic malignancy, dementia, or care home residence.

All analyses were performed using Stata (version 16; StataCorp., College Station, TX). Significance was set at *p* < 0.05.

### Standard Protocol Approvals, Registrations, and Patient Consents

Written informed consent was obtained from patients or assent was acquired from relatives if consent was not possible.

### Data Availability

Requests for data will be considered by Rothwell (peter.rothwell@ndcn.ox.ac.uk).

## Results

Of 2,540 patients ascertained with probable or definite strokes between 2002 and 2017, 48 (1.9%) were excluded from the main analysis because of incomplete data on medical history or NIHSS, mainly due to early death, nonhospitalization, or limitations due to end-of-life care (eFigure 1, links.lww.com/WNL/C911). Of the 2,492 (98.1%) remaining patients (mean/SD age = 74.5/13.9 years; 1,216 male; 2,160 ischemic strokes), 1,402 (56.3%) had at least 1 prior CCI comorbidity and 700 (28.0%) had multimorbidity (≥2 CCI comorbidities; Table 1). The most common comorbidities were previous cancer (13.8%), myocardial infarction (11.5%), and diabetes without end-organ damage (11.4%; Table 2 and eTables 1–3).

**Table 1:**
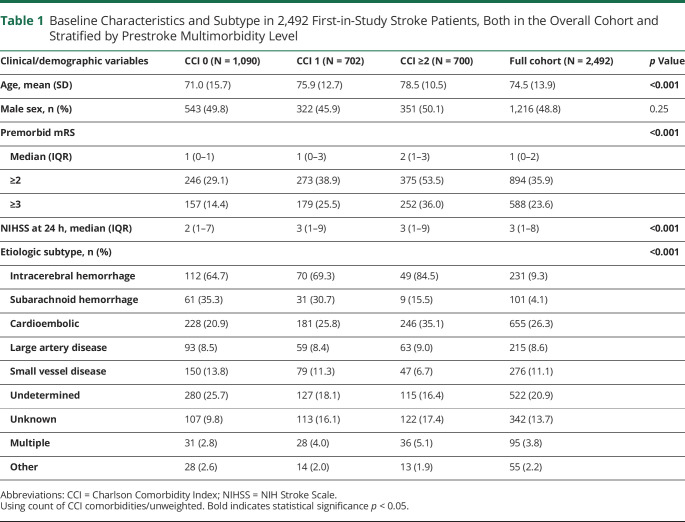
Baseline Characteristics and Subtype in 2,492 First-in-Study Stroke Patients, Both in the Overall Cohort and Stratified by Prestroke Multimorbidity Level

**Table 2:**
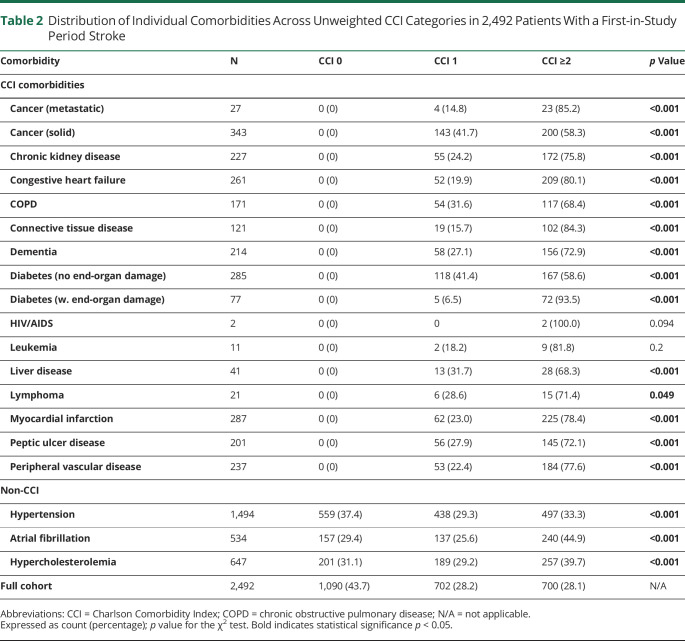
Distribution of Individual Comorbidities Across Unweighted CCI Categories in 2,492 Patients With a First-in-Study Period Stroke

Of patients with ≥2 CCI comorbidities, 375 (53.5%) had a premorbid mRS ≥2 (Table 1). Multimorbidity was strongly associated with premorbid disability (age-adjusted/sex-adjusted odds ratio [aOR] per comorbidity = 1.42, 1.31–1.54; aOR per-weighted CCI point 1.21, 1.14–1.28; both *p* < 0.001).

The proportion of intracerebral hemorrhage (ICH) vs ischemic stroke was lower in patients with multimorbidity (aOR/CCI comorbidity 0.80, 0.70–0.92, *p* < 0.001; eTable 4, links.lww.com/WNL/C911) and remained so after excluding patients with known prior atrial fibrillation (0.83, 0.71–0.96, *p* = 0.016).

The CCI was crudely associated with increased NIHSS at 24 hours (OR per CCI comorbidity 1.11, 95% CI 1.01–1.23 for NIHSS 5–9 vs 0–4; 1.12, 1.03–1.21 for NIHSS ≥10 vs 0–4; linear regression using weighted CCI and NIHSS: β = 0.24, *p* = 0.005; eTables 5–7, links.lww.com/WNL/C911) and in analyses confined to ischemic stroke (OR per CCI comorbidity 1.12, 1.01–1.23 for NIHSS 5–9 vs 0–4, *p* = 0.027; 1.15, 1.06–1.26, for NIHSS ≥10 vs 0–4; *p* = 0.001; β = 0.30, *p* < 0.001; eFigure 2). Associations were similar after exclusion of patients with known prior atrial fibrillation (eTable 8), after stratification by age and sex (eTable 9), and after excluding patients who received thrombolysis or thrombectomy (eTable 10).

**Figure 1:**
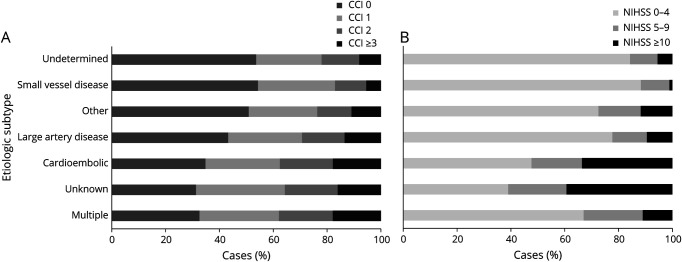
Distribution of the Unweighted CCI (A) and NIHSS Score at 24 Hours (B) Across Different Etiologic Subtypes of Ischemic Stroke Using TOAST Classification Panel A: CCI (counts/unweighted). Panel B: NIHSS. CCI = Charlson Comorbidity Index; NIHSS = NIH Stroke Scale; TOAST = Trial of Org 10172 in Acute Stroke Treatment.

However, CCI differed across TOAST subtypes of ischemic stroke (*p* < 0.001; Figure 1). Compared with patients who had cryptogenic events, comorbidity was greater in those with cardioembolic events (age-adjusted/sex-adjusted OR per CCI comorbidity 1.33, 95% CI 1.19–1.48, *p* < 0.001), large artery disease (aOR 1.20, 1.04–1.38, *p* < 0.01), multiple etiologies (aOR 1.32, 1.01–1.72, *p* < 0.05), and incomplete investigation (TOAST Unknown classification; aOR 1.36, 1.19–1.56, *p* < 0.001; Table 3 and eTables 11–13, links.lww.com/WNL/C911). On analysis of prestroke CCI vs NIHSS at 24 hours within individual TOAST subtypes, no significant associations remained for any subtype or on pooling of the within-subtype associations (Table 4 and eTable 14). Results were similar on linear regression of weighted CCI against NIHSS stratified by subtype (eTables 15 and 16) and in sensitivity analyses designating the 30 patients without brain imaging who died in the community or shortly after arrival in hospital as having an NIHSS ≥10 and having either an ICH or an ischemic stroke of unknown TOAST etiology (eTable 17).

**Table 3:**
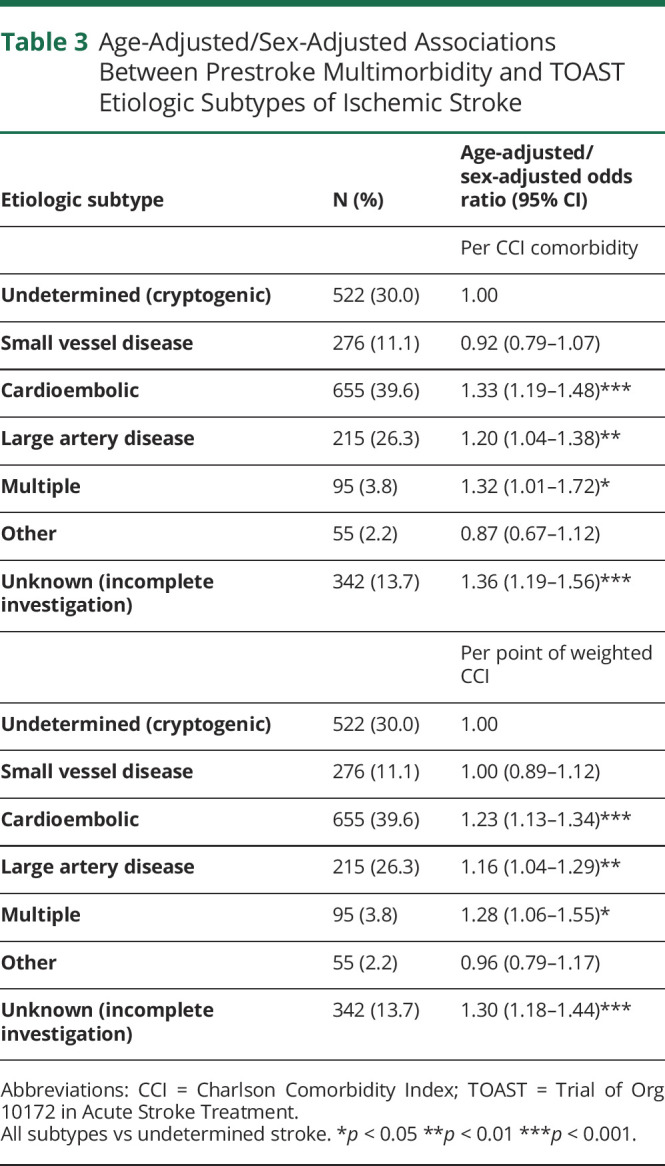
Age-Adjusted/Sex-Adjusted Associations Between Prestroke Multimorbidity and TOAST Etiologic Subtypes of Ischemic Stroke

**Table 4:**
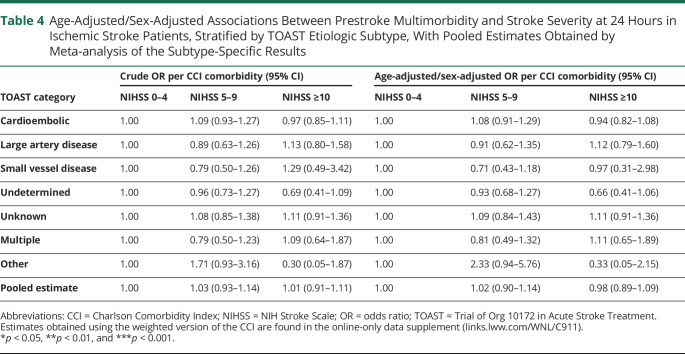
Age-Adjusted/Sex-Adjusted Associations Between Prestroke Multimorbidity and Stroke Severity at 24 Hours in Ischemic Stroke Patients, Stratified by TOAST Etiologic Subtype, With Pooled Estimates Obtained by Meta-analysis of the Subtype-Specific Results

The CCI was modestly predictive of all-cause death at 90 days after adjustment for age, sex, and NIHSS (adjusted hazard ratio [aHR] 1.14, 1.09–1.19, *p* < 0.001) and after further adjustment for premorbid mRS (aHR 1.09, 1.04–1.14, *p* < 0.001; eTable 18, links.lww.com/WNL/C911) and stratification by TOAST subtype (pooled aHR per CCI comorbidity 1.09, 1.04–1.13; *p* < 0.001). Results were similar after excluding patients with metastatic cancer, hematologic malignancy, dementia, or care home residence (eTable 19).

## Discussion

Multimorbidity has been widely reported to be associated with an increased short-term and medium-term mortality after stroke, which might provide some justification for exclusion of patients from randomized controlled trials of certain interventions. However, although some previous studies of the association of multimorbidity with outcome after stroke adjusted for stroke severity, none of the 22 studies included in recent reviews looked specifically at the association between multimorbidity and early stroke severity, and none stratified analyses by etiologic subtype of ischemic stroke.^2,20^ We showed that although multimorbidity was strongly associated with premorbid disability, consistent with studies in the general population,^21-23^ there was no association with greater stroke severity after stratification by etiologic subtype, and there was only a modest association with 90-day mortality after adjustment for severity and prior disability.

One previous study reported analyses of the prognostic value of multimorbidity in ischemic stroke vs ICH,^24^ but we are not aware of previous reports comparing multimorbidity rates between these subtypes or between TOAST subtypes of ischemic stroke. The observed association in our study of multimorbidity with cardioembolic stroke might be expected due to the inclusion of cardiac diseases in the CCI (myocardial infarction, congestive heart failure) and that with large artery disease etiology likely reflects the inclusion of related comorbidities (myocardial infarction, peripheral vascular disease, diabetes) in the CCI. These associations would in turn explain the link between CCI and stroke due to multiple etiologies. The tendency for patients with multimorbidity to receive incomplete investigation (“unknown” TOAST subtype) probably reflects the fact that some comorbidities contraindicate investigations (e.g., contrast-dependent arterial imaging, MRI brain imaging), and investigations might be avoided for compassionate or pragmatic reasons in patients with comorbidities such dementia or metastatic cancer.

Our findings have implications for stroke researchers, clinicians, and policymakers. First, our results underscore the importance of adjusting for premorbid disability and stratifying by etiologic subtype in future research studies of multimorbidity and stroke. Second, given the aging population and predicted rises in rates of multimorbidity, our data suggest that the overall cost and burden of stroke will rise in parallel due to the association with stroke subtypes that tend to be more severe even though multimorbidity seems not be associated with a greater severity of stroke within individual subtypes. Third, this lack of a subtype-specific association of multimorbidity with stroke severity, and the weak association with early mortality after adjustment for confounders, suggests that the presence of multimorbidity per se should not discourage active treatment in routine clinical practice. Previous studies of the association of multimorbidity and poststroke mortality have focused mainly on longer-term mortality, and reports of increased early mortality may have overestimated the association by use of administrative data alone, without the potential to stratify by stroke subtype.^11,24^

Our findings also have implications for the design, performance, and interpretation of trials in patients with stroke. First, our finding that more than a quarter of stroke patients has multiple prior comorbidities highlights the importance of being able to apply trial evidence in clinical decision-making in this group. Second, our finding that multimorbidity was not independently related to stroke severity, and was only weakly associated with short-term mortality, suggests patients with multimorbidity should not be routinely excluded from trials unless there is a specific reason to do so. Third, our finding that approximately half of stroke patients with multimorbidity have an mRS ≥2 highlights 1 important mechanism for exclusion from trials and supports the use of primary outcomes based on ordinal rather than dichotomous analysis of the mRS.^6^ Removal of this barrier to inclusion of patients with premorbid disability leaves little residual justification to exclude patients with multimorbidity either by strict inclusion criteria or by investigators concerned about trial discipline or any adverse effects on power to detect treatment effects. Finally, our finding that multimorbidity is not associated with a greater proportion of ICH vs ischemic stroke should encourage greater inclusion of patients with comorbidities in trials of antithrombotic treatment in prevention of stroke,^7^ particularly if any increased risk of gastrointestinal bleeding can be mitigated.

Our study did, however, have several limitations. First, there is a risk of ascertainment bias whereby patients with multimorbidity may be less likely to present after minor stroke or clinicians may attribute symptoms of minor events to existing comorbidities. However, our case ascertainment aimed to identify all patients who sought medical attention with TIA/stroke symptoms irrespective of the presumptive diagnosis of the clinician who first assessed them. Moreover, residual underascertainment of patients with minor events would have increased the apparent association of multimorbidity with stroke severity. We also attempted to minimize any potential bias in relation to delayed presentation or delayed initial clinical assessment in patients with multimorbidity by assessing the NIHSS at a uniform time point after stroke onset (usually approximately 24 hours). Second, it is possible there was some underascertainment of comorbidities, particularly in patients with aphasia or dementia. However, we also obtained recorded prior comorbidities from all hospital and primary care medical records. Third, our analyses stratified by etiologic subtype of ischemic stroke were underpowered to exclude weak associations with outcome within subtypes. Fourth, potential withdrawal/withholding of care or advanced directives in patients with multimorbidity might bias associations with outcome. However, although we did not collect data on withdrawal/withholding of care or on advanced directives, exclusion of patients most likely to have treatment withheld did not alter our findings. Similarly, we did not measure the intensity of care in stroke patients with multimorbidity, which should be explored in future studies, but sensitivity analyses in relation to receipt of thrombolysis or thrombectomy did not alter our findings. Fifth, our findings should be interpreted in light of the fact that our study population is predominantly Caucasian. Finally, the CCI does not include all comorbidities that might be associated with the severity of stroke. For example, we showed that prior atrial fibrillation was strongly predictive of increased severity. However, excluding patients with prior atrial fibrillation did not alter our findings.

In conclusion, multimorbidity was common in patients with stroke and was associated with premorbid disability, but it was not associated with increased severity of stroke independent of etiologic subtype, or with an increased proportion of ICH, and was only modestly associated with 90-day mortality after adjustment for premorbid disability and stroke severity.

## Glossary

aHR: adjusted hazard ratio
aOR: adjusted odds ratio
CCI: Charlson Comorbidity Index
GP: general practitioner
ICH: intracerebral hemorrhage
mRS: modified Rankin scale
NIHSS: NIH Stroke Scale
OXVASC: Oxford Vascular Study
TIA: transient ischemic attack
TOAST: Trial of Org 10172 in Acute Stroke Treatment
